# Mental Demands at Work and Risk of Dementia

**DOI:** 10.3233/JAD-190920

**Published:** 2020-04-07

**Authors:** Anna Sundström, Daniel Eriksson Sörman, Patrik Hansson, Jessica Körning Ljungberg, Rolf Adolfsson

**Affiliations:** a Department of Psychology, Umeå University, Umeå, Sweden; b Centre for Demographic and Ageing Research (CEDAR), Umeå University, Umeå, Sweden; c Department of Clinical Sciences, Umeå University, Umeå, Sweden; d Department of Human Work Science, Luleå University of Technology, Luleå, Sweden

**Keywords:** Aging, Alzheimer’s disease, cognitive occupation complexity, cognitive reserve, dementia, mental demands at work, vascular dementia

## Abstract

High mental demands at work was examined as a possible protective factor to reduce the risk of dementia in 1,277 initially dementia-free participants, aged 60 years and older. The cohort was followed for a mean of 13.6 years. During follow-up, 376 participants developed all-cause dementia (Alzheimer’s disease = 199; vascular dementia = 145). The association between mental demands at work and dementia was analyzed with Cox hazard models, adjusted for a range of covariates. The results revealed no significant association between mental demands at work and incidence of dementia. Based on the measures used in this study, it was concluded that high mental demands at work may not reduce the risk of dementia later in life.

## INTRODUCTION

Most people spend a considerable amount of time at work; yet, little research exists on how this affects cognitive health. It has been suggested that occupational status (blue collar versus white collar) is associated with incidences of dementia [1, 2]. One proposed explanation for these results is that high-status occupations imply greater cognitive stimulation and provide less likelihood of exposure to adverse factors often connected with low-status occupations [3]. Many researchers have also argued that factors, such as higher education and complex work environments, can help build up a substantial cognitive reserve, resulting in a more efficient cognitive network. This cognitive reserve could, in turn, enable one to compensate for age-related cognitive decline as well as pathological changes, such as dementia, by making better use of alternate networks or recruitment of alternative brain networks [4, 5].

According to the cognitive reserve hypotheses, high mental demands at work would facilitate the development of cognitive skills because individuals would continue to hone their cognitive skills when they are exposed to cognitive challenging environments; further, many studies have also reported support for this hypothesis [6–11]. For example, by using data from the Swedish Twin Registry, Andel and colleagues [6] divided the complexity of work into three categories, including level of working with data (e.g., analyzing), people (e.g., mentoring), and things (e.g., precision working). These researchers found that more complex work with people and data is protective of dementia and Alzheimer’s disease (AD). Moreover, for each additional level of complexity of work with people, the risk of AD was reduced by 22%. Kröger et al. [9] later found that high complexity at work was related also to reduce risk of vascular dementia (VaD), even after adjustment for a number of potential confounders (e.g., family history of dementia, diabetes, and coronary heart disease).

However, not all studies find an association [12]; some of the previous study results may be biased by small sample size [9] or selection bias, such as high age at study entry [7, 8, 10] and use of only prevalent dementia cases instead of incident cases [6]. Thus, the existing evidence of a causal relationship between high mental demands at work and development of dementia remains insufficient. Furthermore, most previous studies within this area have used rather rough measures of occupational complexity (e.g., white-collar/blue-collar, skilled/unskilled) or job classification based on the US Dictionary of Occupational Titles (DOT) [13], including such as complexity of work with data, people, or things (for example, [6, 7]).

This study will examine the impact of high mental demands at work on all-cause dementia, AD, and VaD, in a sample with data from the Betula project, a prospective population-based study with up to 24 years of follow-up. To classify mental demands at work, we use variables from the Occupational Information Network (O*NET) database [14, 15]. O*NET contains data for almost 1,000 occupations; each includes cognitive, interpersonal, physical, and working aspects rated by analysts, incumbents, and occupational experts.

## MATERIAL AND METHODS

### Study sample

The population from which participants were drawn has been describe in detail elsewhere [16, 17]. Briefly, the Betula project started in 1988 and includes six independent samples (S1 through S6) and six test waves (T1 through T6), with five years in between each test wave. All participants were randomly sampled (stratified by age and sex) from the population register in the Umeå municipality, Sweden. Each participant was first contacted via mail and then via telephone to schedule a time for health examination and, the week after, cognitive examination. The health and memory examinations took 1.5 to 2 h for each participant. Exclusion criteria for participating in the Betula project were dementia diseases, mental retardation, severe visual or auditory handicaps, or native language other than Swedish.

### Participants

The present study included data from participants who took part in test wave two (T2) and included individuals from Sample 1–3 (S1–S3). T2 served as baselines for this study because data on O*NET codes were first available at that test occasion. S2 and S3 were assessed for the first time at this test wave, whereas S1 had been tested five years previously. Participants in the present study were in cohorts of 60, 65, 70, 75, 80, and 85 of age at study baseline and consisted of an initial sample of 1,457 participants. Excluded participants were those who had a follow-up time of less than one year from the study baseline (*n* = 17), unknown dementia status at follow-up, e.g., due to moving from the catchment area (*n* = 50), incomplete information on the questionnaire regarding work complexity (*n* = 106), missing data on *APOE* genotype (*n* = 38), education (*n* = 15), smoking (*n* = 2), and alcohol (*n* = 2). This left a final sample for the present analyses of 1,227 participants. In Sweden, normal retirement age is 65 years, thus most participants were either retired or became retirement shortly after study baseline basement.

### Diagnosis of dementia

The diagnosis of dementia was based on analysis of neuropsychological testing, structured interviews conducted by trained nurses, and observations made at each test occasion. In addition, participants with a Mini-Mental State Examination score (MMSE) [18] of 23 points or less, or a score on a composite of cognitive/memory test of ≥1.8 SD below age-adjusted norms, were subject for further evaluation. The dementia diagnoses were based on the DSM IV criteria [19]. The more detailed NINCDS-ADRDA criteria for probable AD [20] and cognitive and neurological symptoms of vascular complications were, in addition, considered for the diagnosis of VaD [21]. Moreover, additionally to the above procedure were evaluations of medical records done every five years.

### Occupational information

Questionnaires about socioeconomic factors, including main lifetime occupation and description of its job characteristics, were completed by the participants at home and returned to the nurse at the later health examination. In cases when participants hade incomplete questionnaires (i.e., missing data), the nurse made sure that the participants completed them. The main occupation obtained was first coded according to Swedish occupational classification, and then linked to the O*NET database (http://www.onetonline.org/) through a recent developed Swedish database where over 8,000 participants occupations received an O*NET classification [22].

The O*NET database contains details of occupational characteristics cover cognitive, interpersonal, and physicals skill requirements, and working conditions. For the purpose of this study, a mental demands at work composite score was created by summing all 21 O*NET variables (1.A.1a–1.A.1.g.2) reflecting level of cognitive ability needed to perform the job, ranging from 0 to 7 (sum score, *M* = 57.99, *SD* = 10.19). The Cronbach’s alpha for the 21 variables included was 0.96, demonstrating very high internal consistency. For the composite score, both skewness (0.24) and kurtosis (0.39) revealed normally distributed data according to guidelines [23].

### Confounders

Potential confounders collected at the baseline examination included age, gender, education (years), smoking (smokers/nonsmokers), alcohol (yes/no, never drank/no had quit), and previous cardiovascular disorders (number of self-reported heart disease, stroke, hypertension, and diabetes over the last five years). Furthermore, the Apolipoprotein E (*APOE*) data were entered as covariate and coded as the presence or absence of the *APOE* *ɛ*4 allele. The *ɛ*2/*ɛ*4 were excluded due to conflicting findings regarding its effects of dementia [24].

### Statistics

Descriptive analyses of baseline characteristics are presented as proportions and mean values with standard deviations. Separate multivariate-adjusted Cox hazard models were used to examine the association between mental demands at work and dementia. Time to event was defined as date of first entry into the study to the date of dementia, death, or date of final follow-up, depending on which comes first. The results were presented as hazard ratios (HRs) with 95% confidence intervals (CIs) and considered as significant at *p* < 0.05. All statistical analyses were performed using the Statistical Package for Social Science (SPSS) Version 26.0.

## RESULTS

Among the 1,277 participants, the mean age was 70.2 (±7.5) at baseline; 57% were women and 43% were men. There were 376 incidences of dementia (AD = 199; VaD = 145) over the follow-up period. Mean follow-up time was 13.6 (±7.2) years for those who remained dementia-free, and 9.6 (±5.7) years for those who received a dementia diagnosis (9.3 years for AD and 10.0 years for VaD). Baseline characteristics for all participants, according to dementia status, are shown in Table 1.

**Table 1:** Study participant baseline characteristics

Characteristic	No dementia *n* = 851	Incident dementia during follow-up		
		Dementia *n* = 376	AD *n* = 199	VaD *n* = 142
Age, mean±SD	69.78 (7.84)	71.00 (6.78)	71.16 (6.70)	70.92 (6.70)
Female, n (%)	447 (52.5)	252 (67.0)	152 (76.4)	82 (57.7)
Education (y), mean±SD	8.11 (3.18)	8.26 (3.57)	8.14 (3.42)	8.55 (3.86)
Current smoking, n (%)
Yes	384 (45.1)	157 (41.8)	78 (39.2)	62 (43.7)
No	467 (54.9)	219 (58.2)	121 (60.8)	80 (56.3)
Alcohol use, n (%)
Yes	589 (69.2)	249 (66.2)	130 (65.3)	98 (69.0)
No, never drank or have quit	262 (30.8)	127 (33.8)	69 (34.7)	44 (31.0)
Number of previous cardiovascular disorders, mean±SD	0.98 (0.87)	0.92 (0.86)	0.77 (0.81)	1.13 (0.93)
*APOE* *ɛ*4 genotype, n (%)
Carriers	182 (21.4)	147 (39.1)	90 (45.2)	49 (34.5)
Non-carriers	669 (78.6)	229 (60.9)	109 (54.8)	93 (65.5)
Mental demands at work
Composite score, sum±SD	58.21 (9.98)	57.48 (10.64)	57.38 (10.70)	57.97 (10.54)
Category flexibility, mean±SD	3.01 (0.48)	3.00 (0.48)	3.00 (0.47)	3.02 (0.50)
Deductive reasoning, mean±SD	3.30 (0.62)	3.29 (0.61)	3.28 (0.60)	3.32 (0.63)
Fluency of ideas, mean±SD	2.67 (0.72)	2.66 (0.75)	2.64 (0.75)	2.72 (0.75)
Inductive reasoning, mean±SD	3.17 (0.60)	3.15 (0.62)	3.17 (0.65)	3.15 (0.62)
Information ordering, mean±SD	3.15 (0.48)	3.10 (0.49)	3.10 (0.49)	3.12 (0.48)
Mathematical reasoning, mean±SD	2.27 (0.91)	2.19 (0.91)	2.18 (0.89)	2.23 (0.94)
Originality, mean±SD	2.63 (0.76)	2.61 (0.77)	2.59 (0.77)	2.66 (0.77)
Flexibility of closure, mean±SD	2.63 (0.51)	2.58 (0.53)	2.59 (0.54)	2.57 (0.51)
Memorization, mean±SD	2.17 (0.54)	2.15 (0.62)	2.15 (0.63)	2.20 (0.59)
Number facility, mean±SD	2.37 (0.76)	2.32 (0.79)	2.32 (0.79)	2.34 (0.78)
Oral comprehension, mean±SD	3.79 (0.52)	3.79 (0.52)	3.80 (0.51)	3.80 (0.56)
Oral expression, mean±SD	3.69 (0.62)	3.72 (0.62)	3.72 (0.60)	3.73 (0.66)
Perceptual speed, mean±SD	2.52 (0.45)	2.45 (0.47)	2.44 (0.49)	2.45 (0.45)
Problem sensitivity, mean±SD	3.35 (0.64)	3.34 (0.66)	3.34 (0.69)	3.37 (0.63)
Selective attention, mean±SD	2.81 (0.32)	2.79 (0.36)	2.79 (0.37)	2.79 (0.34)
Spatial orientation, mean±SD	0.79 (0.91)	0.63 (0.83)	0.59 (0.81)	0.67 (0.85)
Speed of closure, mean±SD	2.21 (0.53)	2.15 (0.56)	2.15 (0.58)	2.18 (0.53)
Time sharing, mean±SD	2.55 (0.36)	2.55 (0.42)	2.55 (0.44)	2.56 (0.38)
Visualization, mean±SD	2.60 (0.67)	2.47 (0.62)	2.43 (0.61)	2.50 (0.62)
Written comprehension, mean±SD	3.40 (0.68)	3.39 (0.70)	3.39 (0.68)	3.43 (0.75)
Written expression, mean±SD	3.13 (0.80)	3.13 (0.85)	3.14 (0.83)	3.16 (0.90)

To examine the association between mental demands at work and dementia, Cox proportional regression analysis was conducted, adjusted first for age and gender, then additional adjustments were made for education, smoking, alcohol, previous cardiovascular disorders, and *APOE* genotype.

The results revealed no association of mental demands at work with reduced risk of all-cause dementia, AD, or VaD, in either a model adjusted for age and gender or in a model in which adjustments were made for a range of covariates (see Table 2).

**Table 2:** Cox proportional hazard regressions models for the association between mental demands at work and incident of dementia, AD, and VaD

	Dementia diseases *n* = 1 227, 376 demented		AD *n* = 1050, 199 demented		VaD *n* = 993, 142 demented	
	Model 1	Model 2	Model 1	Model 2	Model 1	Model 2
Mental demands at work	0.99 (0.99–1.01)	0.99 (0.98–1.01)	1.00 (0.99–1.02)	1.00 (0.98–1.02)	1.00 (0.98–1.02)	0.99 (0.97–1.01)
Age	1.08 (1.07–1.10)^***^	1.09 (1.07–1.11)^***^	1.08 (1.06–1.10)^***^	1.09 (1.07–1.12)^***^	1.08 (1.06–1.11)^***^	1.09 (1.07–1.12)^***^
Woman^a^	1.31 (1.04–1.64)^*^	1.29 (1.02–1.63)^*^	2.24 (1.59–3.15)^***^	2.23 (1.57–3.18)^***^	0.97 (0.68–1.38)	0.92 (0.64–1.33)
Education		1.02 (0.99–1.06)		1.00 (0.95–1.05)		1.05 (1.00–1.11)
Smoking^b^		1.08 (0.87–1.35)		1.10 (0.81–1.48)		1.05 (0.73–1.50)
Alcohol consumption^c^		0.94 (0.75–1.18)		0.95 (0.70–1.29)		1.03 (0.71–1.50)
Prior cardiovascular disorders		1.15 (1.02–1.30)^*^		0.89 (0.75–1.08)		1.54 (1.27–1.87)^***^
*APOE*^d^		2.31 (1.87–2.86)^***^		3.02 (2.27–4.02)^***^		2.25 (1.58–3.21)^***^

Model 1 was adjusted for age and gender. Model 2 was adjusted for age, gender, education, smoking, alcohol use, previous cardiovascular disorders, and *APOE* genotype. ^a^Male is the reference group. ^b^No smoking is the reference group. ^c^Never drank or have quit is the reference group. ^d^*APOE* *ɛ*3 is the reference group. ^*^p < 0.05, ^**^p < 0.01, ^***^p < 0.001.

Results remained similar to the main analyses when we excluded participants with onset of dementia within five years, to reduce the risk of preclinical dementia symptoms affecting the results (results not shown).

## DISCUSSION

In this population-based cohort study, with up to 24 years of follow-up, we found no evidence that higher levels of mental demands at work were associated with neither all-cause dementia nor dementia subtypes (i.e., AD or VaD). Sensitivity analysis, where those receiving a dementia diagnosis within five years from baseline were excluded, did not change the results.

The results of the present study are in contrast with earlier findings, suggesting an association between work complexity and dementia [6–11], although some researchers report findings similar to ours [12]. Our results also correlate fairly well with research examining cognitive change in old age and those who find only an association between level of cognitive performance and occupational complexity at baseline, but no association between occupational complexity and cognitive change [25, 26]. The discrepancy between findings can be explained by the heterogeneity in different study designs (cross-sectional versus longitudinal), definitions of occupational complexity, data collection (retrospective versus prospective), statistical methods, and adjustment for different covariates.

A major strength with this study is that it is based on population-based data from northern Sweden, which has a relatively homogeneous population in terms of socioeconomic standards and whereas all habitants have access to free healthcare, thereby reducing the risk that socioeconomic factors confound the results. Additional strengths include random sampling of the participants (stratified by age and gender) from the population register, the prospective design, long follow-up time, and the possibility to adjust for a range of potential confounders.

Despites its many strengths, this study has important limitations. Although O*NET data are considered a validated measurement of occupation characteristics, the categorization of mental demands at work for each occupation are on a group level and an account is not taken of the individual’s own experience of their profession, for example, a specific occupation may not provide exactly the same cognitive demands for all persons. In addition, we used the O*NET database from 2002 and the demands of an occupation at that time may differ from when the population in this study was working, which could be considered a threat to the validity of the occupation data. Furthermore, in the O*NET database there is no coding for homemakers; therefore, these persons were excluded from the analyses.

The findings of this study suggest that high mental demands at work does not appear to protect against or reduce the risk of dementia; this study, therefore, cannot be considered to support the hypothesis of cognitive reserve [4, 5].
